# Refining the South Asian Origin of the Romani people

**DOI:** 10.1186/s12863-017-0547-x

**Published:** 2017-08-31

**Authors:** Bela I. Melegh, Zsolt Banfai, Kinga Hadzsiev, Attila Miseta, Bela Melegh

**Affiliations:** 1 University of Pecs, Szentagothai Research Centre, Ifjusag Road 20, Pecs, H-7624 Hungary; 2 Department of Medical Genetics, University of Pecs, Clinical Centre, Szigeti Road 12, Pécs, H-7624 Hungary; 3 Department of Laboratory Medicine, University of Pecs, Medical School, Szigeti Road 13, Pecs, H-7624 Hungary

## Abstract

**Background:**

Recent genetic studies based on genome-wide Single Nucleotide Polymorphism (SNP) data further investigated the history of Roma and suggested that the source of South Asian ancestry in Roma originates most likely from the Northwest region of India.

**Methods:**

In this study, based also on genome-wide SNP data, we attempted to refine these findings using significantly larger number of European Roma samples, an extended dataset of Indian groups and involving Pakistani groups into the analyses. Our Roma data contained 179 Roma samples. Our extended Indian data consisted of 51 distinct Indian ethnic groups, which provided us a higher resolution of the population living on the Indian subcontinent. We used in this study principal component analysis and other ancestry estimating methods for the study of population relationships, several formal tests of admixture and an improved algorithm for investigating shared IBD segments in order to investigate the main sources of Roma ancestry.

**Results:**

According to our analyses, Roma showed significant IBD sharing of 0.132 Mb with Northwest Indian ethnic groups. The most significant IBD sharings included ethnic groups of Punjab, Rajasthan and Gujarat states. However, we found also significant IBD sharing of 0.087 Mb with ethnic groups living in Pakistan, such as Balochi, Brahui, Burusho, Kalash, Makrani, Pashtun and Sindhi.

**Conclusion:**

Our results show that Northwest India could play an important role in the South Asian ancestry of Roma, however, the origin of Romani people might include the area of Pakistan as well.

**Electronic supplementary material:**

The online version of this article (10.1186/s12863-017-0547-x) contains supplementary material, which is available to authorized users.

## Background

The Romani people (Roma), living mainly in Europe with an approximate 10–15 million number of individuals, are a very diverse and unique population [1]. With smaller population sizes, they also can be found in the Caucasus region, Middle East and have also Pan-American populations.

The Roma belong to the Indo-European language family, speak more than 60 dialects of Romani language and do not have a single convention for writing. Because of their diverse nature, they do not have a written history, therefore experts can only infer to their history through linguistics, historical records of other nations they contacted with and through genetic investigations.

Cultural, linguistic and historical studies have suggested that the Roma are originating from South Asia and migrated towards Europe between the 5th and 10th century [2]. Their possible migration route could include the Caucasus and the Anatolian Peninsula [2, 3]. Roma were driven from the Balkans into Europe by the Ottoman conquest campaigns in the 11th and 12th centuries and became widespread throughout Europe in the 15th century already [2]. Most of the Roma people live currently mainly in the Balkans, the Iberian Peninsula and also in East-Central Europe [4, 5].

Studies investigating Roma culture revealed significant similarities between Roma and Indian culture including the caste system and endogamic habits that means exclusive marriage within Roma sub-ethnic groups (clans) [2]. Linguistic studies suggested that the most closely related languages to the Roma language are certain spoken languages in Northwest India such as Punjabi and Kashmiri, and indicated also a link between the Central Indian Hindi and Roma language [6, 7].

Genetic studies based on Y-chromosome markers and mitochondrial DNA confirmed the South Asian origin of Roma. Y-chromosome marker M82 (H1a) and mtDNA haplogroups M5a1, M18 and M35b, which are characteristics of South Asian ancestry, are typical in Roma populations [8, 9]. However, studies based on Y-chromosome and mtDNA are contradicting with each other. A study investigating the Y-chromosome suggested that Roma are originating from South India, while mtDNA-based studies concluded that Indian ancestry of Roma originating from Northwest India [10].

While studies based on Y-chromosome markers or mtDNA provided valuable information about Roma history, the limitations of this investigation are clear, since they provide information only about the paternal or maternal lineages, and cannot show us the whole genealogy of Roma as it is. Study of autosomal data provides the simultaneous analysis of multiple genealogies, which can provide additional information about the history of Roma. Recent studies, based on genome-wide autosomal single nucleotide polymorphism data, were able to determine the source of South Asian and European ancestry of Roma and the fact that Roma are an admixed ethnic group with West Eurasian and South Asian ancestry [11, 12]. These studies estimated the proportion of West Eurasian ancestry of Roma and also the date of European gene flow that shaped the Roma population into its current state. Founder events that can be held responsible for the high level of genome-wide homozygous-by-descent segments estimated in Roma were also investigated. Both studies place the origin of Romani people to the Northwest region of India inhabited mainly by Punjabi, Gujarati and Kashmiri Pandit.

Here we analyzed whole genome SNP array data from a set of 179 European Roma samples and an extended set of Indian samples. We attempted to refine these findings by applying a dataset containing higher number of Roma samples, which could model the Roma population living in Europe more accurately. Utilization of significantly higher number of Indian ethnic groups allowed also to reinvestigate the source of South Asian ancestry by providing a much higher resolution of the Indian population. A recent study reported that the source of Indian ancestry of Roma is most likely Northwest India [11]. In this study, ethnic groups living also in Pakistan (Pashtun or Pathan and Sindhi) were applied besides Indian ethnic groups to represent the population of Northwest India. In order to investigate the extent of Pakistani involvement of the South Asian ancestry of Romani people, we included also seven Pakistani groups in our tests.

## Methods

### Datasets

We used in this study upon request available and in international collaboration collected and genotyped datasets. Our Roma sample collection consisted of two datasets. Twenty-seven Roma samples were collected and genotyped in international collaboration [11], which was merged with further samples of 152 Roma individuals obtained as an upon request available dataset. [12]. Both set of samples were genotyped using Affymetrix 1 M chip. Based on PCA and clustering methods, we removed Roma individuals from the merged Roma dataset, which showed significant admixture with non-Roma Europeans. The merged dataset contained 158 Roma samples featuring 599,472 autosomal SNPs. These data were merged with datasets from five other sources, including the International Haplotype Map Phase 3 (HapMap) (*n* = 1115 from 11 populations genotyped on Illumina Human1M and Affymetrix 1 M platforms), the CEPH-Human Genome Diversity Panel (HGDP) (*n* = 1043 from 52 populations, 660,918 SNPs genotyped on Illumina 650 Y array), the authorized access requiring Population Reference Sample (POPRES) (*n* = 4077 from 57 populations, 453,617 SNPs genotyped on Affymetrix 500 K platform) [13] and an upon request available merged data containing Indian samples from two previous studies (*n* = 378 from 51 groups, 494,863 SNPs genotyped on Affymetrix 1 M and Illumina 650 K arrays) [11, 14].

**Fig. 1:**
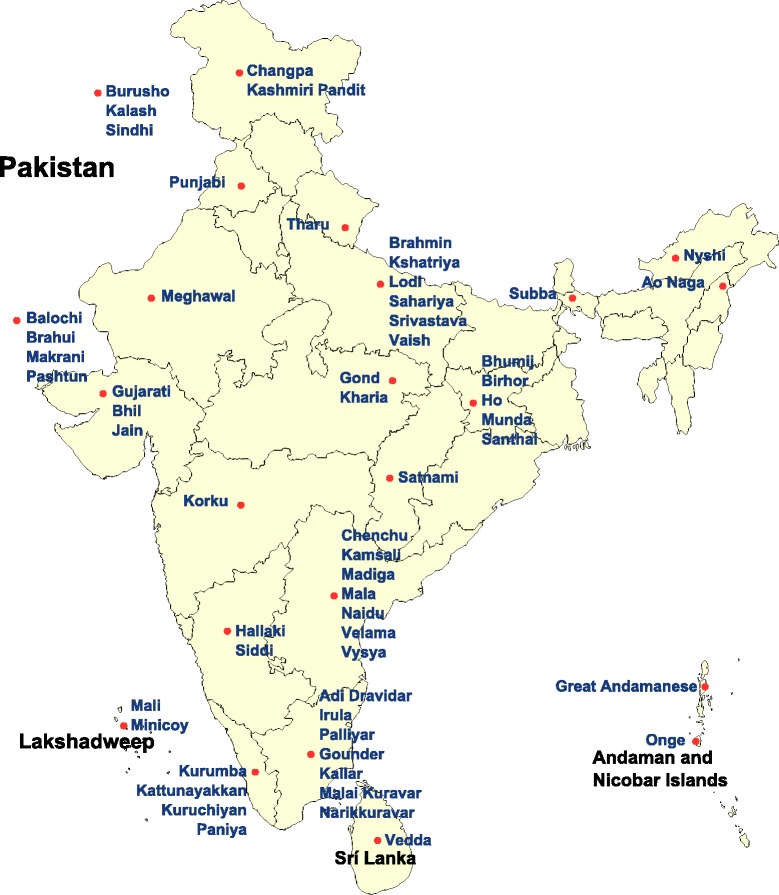
The map of India and the surrounding area. The location of investigated South Asian groups

All groups of the HGDP and HapMap data were used in certain tests. The Indian groups Punjabi and Gujarati and samples from European countries were added to our analyses from the POPRES dataset. Following the preliminary analyses, 42 Indian groups were used from the combined Indian data. (Fig. 1) Depending on the analysis, we included different number of populations from these sources.

### Population structure analysis and F_st_ calculations

To study the relationship of Roma with South Asian populations and to place the Roma in an Eurasian perspective, we created a merged dataset of Roma, CEU (Utah residents with Northern and Western European ancestry from the CEPH Collection) and CHB (Han Chinese in Beijing, China) populations from the HapMap data, two Pakistani groups (Pashtun, Sindhi) from HGDP data, the Indian groups Punjabi and Gujarati from POPRES data and 42 populations from the Indian data (*n* = 704 from 56 populations and 62,509 SNPs). Preliminary ancestry analyses showed that Siddis have significant recent African related ancestry, therefore Siddi samples were removed from the Indian data applied in our analyses. Changpa, Ao Naga, Nyshi, Subba, Korku and Mala groups showed complex, mainly East Asian related ancestry in the preliminary analyses and were also removed from the Indian data. SNPs, which are in strong LD can affect Principal Component Analysis (PCA) and ADMIXTURE analyses, therefore we thinned the marker set of the merged dataset using PLINK v1.07 in order to eliminate background LD. We set the pairwise genotypic correlation variable r^2^ to 0.3. Size of the sliding window was 50 SNPs with a sliding of five SNPs at a time. The thinned datasets contained 46,258 SNPs, respectively. We used SMARTPCA [15] to perform PCA and to compute F_st_ values. Clustering analysis was carried out using ADMIXTURE [16]. We made a cross-validation error check with K = 2 to K = 8 hypothetical ancestral groups to find the K-value that fits appropriately the investigated data.

We applied also TreeMix 1.13, which estimates a maximum likelihood (ML) tree of investigated populations based on genome-wide allele frequency data [17]. The output of the software can be plotted as an ML graph visualizing population splits, admixture events and optionally can also estimate and show probable past migration processes. First we computed the TreeMix graph of HapMap populations and Roma samples in order to attempt to place the Romani people in a worldwide context. Similarly to previous population structure and ancestry analyses, the merged HapMap and Roma data were also pruned using the PLINK Software package to eliminate background LD. The r^2^ was set to 0.1 with a window size of 50 SNPs sliding five SNPs at a time. After the pruning process, this merged dataset contained 83,807 SNPs. We used Yoruba samples (YRI) as root population, set the SNP block size (−k option) to 3000, and allowed five migration events in the tree using the -m option. Another TreeMix analysis was also performed applying Eurasian populations, consisting of Roma, CEU, the outgroup CHB and ethnic groups form India and Pakistan (Pashtun, Sindhi). In order to provide a simple graph, unlike PCA and ADMIXTURE analyses, we applied only a small number of Indian ethnic groups representing the major regions of India (Northern India: Brahmin, Gujarati and Punjabi; Southern region of India: Madiga, Narikkuravar and Vysya; Andaman and Nicobar Islands: Onge). Background LD based SNP pruning with PLINK was applied using the same settings as described previously at PCA and ADMIXTURE analysis. The Roma, HapMap and Indian merged dataset contained 46,501 SNPs. We used CHB as the root population of the TreeMix analysis and set the SNP block size parameter to 1000. We added four migration events to the tree with the -m option of TreeMix.

### Formal test of admixture - estimating ancestry proportion

We applied the F_4_ Ratio Estimation method of ADMIXTOOLS 1.1 Software Package [18] in order to estimate the genome-wide proportion of West Eurasian and South Asian ancestries of Roma. Using F_4_ Ratio Estimation, we investigated the ratio of f4(TSI, CHB, Roma, Onge)/f4(TSI, CHB, CEU, Onge), which gives the excess of West Eurasian ancestry in Roma compared to South Asian ancestry, represented here by the Onge. According to the literature Onge do not have recent West Eurasian ancestry. [19] We also computed the proportion of West Eurasian ancestry in Roma compared to all other Indian groups, however these populations have varying extent of West Eurasian ancestry. We created for this analysis a new merged dataset containing Roma, HapMap and Indian data.

### Beagle refined IBD analyses

We used the Refined IBD algorithm of Beagle 4 [20] to identify the source of European and South Asian ancestries of Roma and to estimate the extent of homozygous by descent segments present in Romani people. We created two distinct datasets for these analyses.

For investigating the source of European and South Asian ancestries, we created a merged dataset consisting of Roma, Indian, Pakistani ethnic groups and European populations. European populations were extracted from the POPRES data. The merged dataset contained the following European populations: *North European* (Norwegian, Swedish), *South European* (Alban, Greek, Italian, Macedonian, Portugal and Spanish), *West European* (Austrian, Belgian, British, Dutch, French, German), *East European* (Russian and Ukrainian) and *Central European* (Bosnian, Croatian, Czech, Hungarian, Polish, Romanian, Serbian, Slovakian) samples. Indian and Pakistani populations were extracted from the Indian, POPRES and HGDP data. Our merged dataset contained the following South Asian (Indian and Pakistani) populations: *North Indian* (Kashmiri Pandit, Punjabi, Tharu, Brahmin, Kshatriya, Vaish), *Northwest Indian* (Meghawal, Gujarati, Bhil, Jain), *Northeast Indian* (Bhumij, Birhor, Ho, Munda, Santhal, Lodi, Sahariya, Srivastava), *Central Indian* (Gond, Kharia, Satnami), *South Indian* (Kurumba, Kattunayakkan, Kuruchiyan, Paniya, Vedda, Adi-Dravidar, Gounder, Kallar, Malai Kuravar, Narikkuravar, Palliyar), *Southwest Indian* (Hallaki, Mali, Minicoy), *Southeast Indian* (Chenchu, Kamsali, Madiga, Naridu, Velama, Vysya), *Andaman and Nicobar Islands* (Great Andamanese, Onge), *Pakistani* (Balochi, Brahui, Burusho, Kalash, Makrani, Pashtun, Sindhi). The merged dataset contained *n* = 1833 individuals and 53,928 SNPs.

Major alleles was set as A1 allele with PLINK v1.07 [21] and the dataset in binary PLINK format was converted to Variant Call Format 4.1 using the PLINK/SEQ v0.10 [22] package. We set the minimum IBD segment length to 3 cM, chose the IBD trim parameter setting to 10, and applied an IBD scale parameter according to the recommended $\sqrt{n/100}$ setting. The recommended setting applies if the dataset contains more than 400 individuals. Otherwise an IBD scale value of 2 is recommended for the analyses [20]. We left all other parameters on its default setting.

We used the output of Beagle 4 to compute an average pairwise IBD sharing between populations I (Roma) and J (European or South Asian groups).

$$ Average\kern0.3em pairwise\kern0.3em IBD\kern0.3em sharing=\frac{\sum_{i=1}^n{\sum}_{j=1}^m{IBD}_{ij}}{n\cdot m} $$

where IBD_ij_ is the length of IBD segment shared between individuals i and j and n, m are the number of individuals in population I and J [23].

### Homozygozity by descent analyses

Besides estimating identity-by-descent segments between pairs of individuals, Refined IBD is also simultaneously seeks shared segments of homozygosity-by-descent (HBD), which allows us to estimate the extent of homozygous DNA segments derived from a single source in case of Romani people. In the HBD analyses, we used the same settings of Refined IBD as in IBD analyses. For estimating the extent of HBD of Roma and other worldwide populations, we created a merged dataset containing Roma samples and all HGDP data (*n* = 1190 from 52 populations, 294,740 SNPs). We computed the overall length of HBD segments in Roma and worldwide HGDP populations and plotted as a function of the number of estimated HBD segments. The regional groups of HGDP populations were the following: *European* (Adygei, Basque, French, North Italian, Orcadian, Russian, Sardinian, Tuscan), *West Asian* (Bedouin, Druze, Palestinian), *Central and South Asian* (Balochi, Brahui, Burusho, Hazara, Kalash, Makrani, Pashtun, Sindhi, Uyghur), *East Asian* (Cambodian, Dai, Daur, Han Chinese, Hezhen, Japanese, Lahu, Miao, Mongolian, Naxi, Orogen, She, Tu, Tujia, Xibo, Yakut, Yi), *African* (Bantu, Biaka Pygmy, Mandeka, Mbuti Pygmy, Mozabite, San, Yoruba) *Native American* (Colombian, Karitiana, Maya, Pima, Surui), *Oceanian* (Melanesian, Papuan).

### Estimating the date of admixture

In order to infer the date of the gene flow between Roma and West Eurasians, we applied the ROLLOFF algorithm included in the ADMIXTOOLS 1.1 Software Package and ALDER 1.03 [24], which is based on the same principle but has many advancements compared ROLLOFF. Both algorithm utilizes the decay of linkage disequilibrium (LD) caused by an admixture event to estimate the time of population admixture. The algorithms compute SNP correlations in an admixed target population and weights the correlations by the allele frequency difference in ancestral populations, which serve as reference populations to the algorithm. These results are sensitive to admixture LD, and the algorithms use allele frequency information in the ancestral populations to amplify the signal of LD caused by the admixture which helps filtering out background LD. Compared to ROLLOFF, ALDER provides more sophisticated weighted LD statistics, has the ability to totally avoid biased estimates caused by background LD and can obtain unbiased statistics by using the target population itself as reference.

To estimate the date of admixture between Roma and West Eurasians we created a merged dataset containing HapMap and Indian data. We used CEU, TSI and Onge as reference populations and the Roma data as the target populations both in ROLLOFF and ALDER. We ran also separate 2-reference tests with ALDER to obtain weighted LD values individually for the tests with reference populations Onge-CEU and Onge-TSI.

## Results

### Ancestry analysis of Roma

We implemented Principal Component Analysis (PCA) using SMARTPCA and the clustering software ADMIXTURE to study the relationship of Roma to Europeans (the HapMap population CEU) and to South Asians. We used Indian groups in these tests, which have mainly West Eurasian and South Asian related ancestries. Populations with significant African or East Asian ancestries were removed from these data based on preliminary ancestry analyses, as we reported in the Materials and Methods section. We used in the PCA and ADMIXTURE analyses 42 Indian groups from the Indian dataset, two Indian groups (Punjabi and Gujarati) from POPRES data and two Pakistani groups (Pashtun, Sindhi) from the HGDP data.

**Fig. 2:**
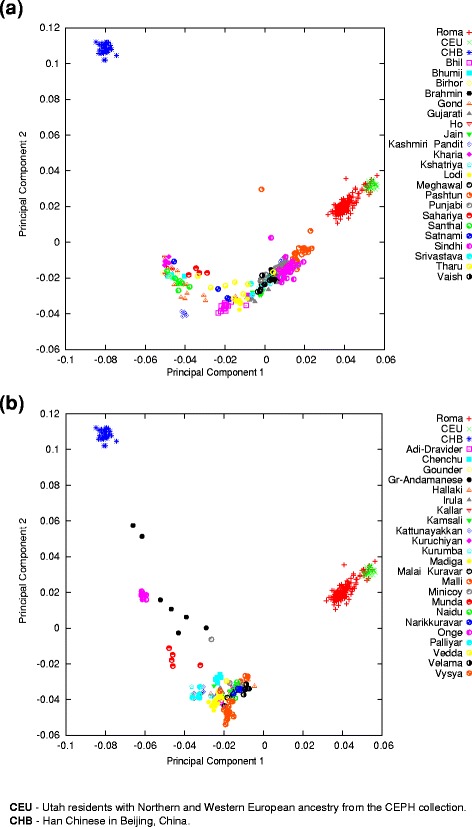
Relationship of Roma to European and South Asian Populations. The principal component analysis results were plotted on the two principal components with the highest eigenvalue. One symbol represents one individual. **a** Relationship of Roma to European and North Indian groups. **b** Relationship of Roma to European and South Indian groups. Separating North and South Indian populations have only practical purpose in order to give a better overview. Both graphs are the result of the same PCA

The PCA result shows a cline where South Asian groups and Roma fall with various relatednesses between Europeans and indigenous groups of the Andaman and Nicobar Islands (Onge and Great Andamanese) (Fig. 2a, b). The Roma are on this cline between the Europeans and South Asians, but are closer to the European samples. Pakistani groups are the closest South Asian groups to the Roma.

**Fig. 3:**
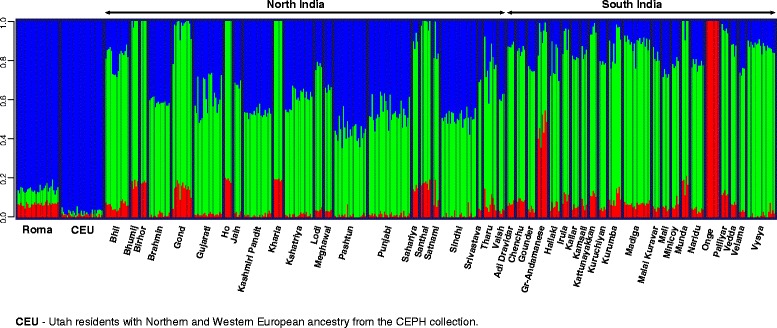
ADMIXTURE analysis of Roma, Europeans and South Asian populations. ADMIXTURE analysis results with K = 3 hypothetical ancestral groups. Each column represents one individual, each column group refers to a certain ethnic group

We applied the model-based ancestry estimation method ADMIXTURE to support the findings of PCA. ADMIXTURE analysis was carried out using K = 2 to 8 hypothetical ancestral groups (Additional file 1). ADMIXTURE analysis showed similar results to PCA at K = 3 hypothetical ancestral groups (Fig. 3). The cline between Europeans and Onge can also be observed on the ADMIXTURE graph. The proportion of West Eurasian ancestry varies greatly within distinct South Asian ethnic groups. The ADMIXTURE analysis results show that Onge do not have recent admixture with West Eurasian populations.

We computed also the pairwise average allele frequency differentiation (F_st_) values with SMARTPCA. We observed that Roma have the lowest F_st_ with European populations and have similarly low F_st_ with Northwest Indian (Gujarati, Punjabi) and Pakistani (Pashtun, Sindhi) populations (Table 1). F_st_ calculations included the HapMap population TSI in order to represent all available European HapMap samples in this test.

**Table 1:** Relationship of Roma to Europeans and South Asians based on pairwise average allele frequency differentiation estimations

North India and Pakistan		South India		Europe	
Bhil	0,030	Adi-Dravidar	0,035	CEU	0,016
Bhumij	0,054	Chenchu	0,081	TSI	0,013
Birhor	0,067	Gounder	0,028		
Brahmin	0,030	Great-Andamanese	0,072		
Gond	0,042	Hallaki	0,033		
Gujarati	0,019	Irula	0,044		
Ho	0,053	Kallar	0,034		
Jain	0,029	Kamsali	0,033		
Kashmiri Pandit	0,027	Kattunayakkan	0,053		
Kharia	0,054	Kuruchiyan	0,030		
Kshatriya	0,025	Kurumba	0,034		
Lodi	0,029	Madiga	0,041		
Meghawal	0,024	Malai Kuravar	0,053		
Narikkuravar	0,070	Mali	0,032		
Pashtun	0,014	Minicoy	0,031		
Punjabi	0,016	Munda	0,047		
Sahariya	0,042	Naridu	0,032		
Santhal	0,046	Onge	0,151		
Satnami	0,034	Palliyar	0,054		
Sindhi	0,017	Vedda	0,088		
Srivastava	0,024	Velama	0,030		
Tharu	0,025	Vysya	0,047		
Vaish	0,018				

**Fig. 4:**
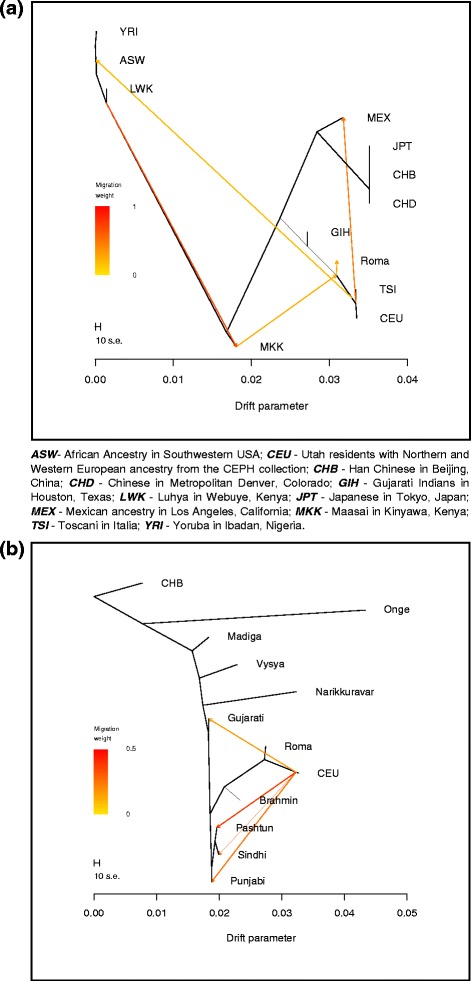
Maximum likelihood tree of Europeans and South Asians. **a** Place of Roma on the ML tree using worldwide HapMap populations. **b** Relationship of Roma with European and Indian populations according to the ML tree constructed with TreeMix

Besides PCA and clustering analyses we applied also the method of the TreeMix algorithm in order to place Romani people on a tree based on a maximum likelihood estimation approach. First we estimated the relationship of Roma with worldwide HapMap populations (Fig. 4a). This analysis show that GIH (Gujarati Indians in Houston, Texas), representing here the South Asians, and Roma fall the same branch as the European populations CEU and TSI (Toscani in Italy), showing that both populations have recent West Eurasian ancestry. The second TreeMix analysis show similar results regarding the relationship of Europeans and Indians (Fig. 4b). Indian populations show various extent of West Eurasian ancestry and also show significant gene flow from West Eurasia to North India. The algorithm placed the Romani people the closest to Europeans, as they have the greatest extent of West Eurasian ancestry from the investigated populations, which can be related to South Asia.

To estimate the proportion of the contribution of West Eurasian ancestry in Roma, we applied the F_4_ Ratio Estimation algorithm from the ADMIXTOOLS Software Package. We used CEU to represent the West Eurasian ancestry of Roma. It is important to note that formal tests of admixture cannot distinguish between West Eurasian populations, usage of other West Eurasian populations, e.g. groups from the Caucasus region, Middle East, Central Asia or European populations other than CEU would provide similar results as applying the CEU group. In our setup, Onge represented the South Asian ancestry component, which do not possess West Eurasian ancestry [19]. Applying F_4_ Ratio Estimation on this setup, our results showed that Roma have on average 81.08 +/− 0.53% West Eurasian related ancestry. D-statistics results, which support that Roma have both West Eurasian and South Asian ancestry are available in Additional file MOESM2. F4 Ratio Estimation results conducted with the usage of other South Asian groups featuring various extent of recent West Eurasian ancestry are shown in Additional file 3. Residuals fit of the TreeMix graphs are shown on Additional file 4.

### Estimating the date of European admixture in Roma

**Fig. 5:**
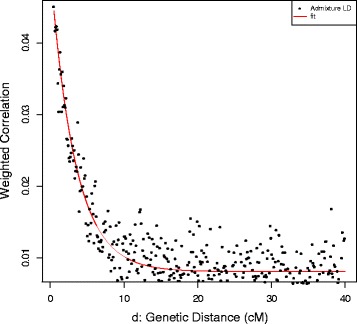
ROLLOFF analysis results. Estimating the date of admixture of Roma with Europeans

**Fig. 6:**
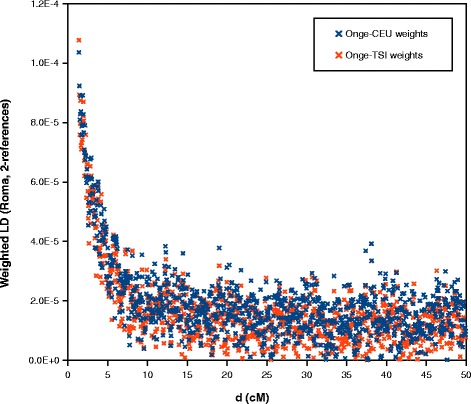
ALDER analysis results. Weighted LD values computed in two separate 2-reference runs (Onge-CEU and Onge-TSI)

We applied ROLLOFF from the ADMIXTURE Software Package to infer the date of gene flow between Roma and West Eurasians. We used CEU and TSI as the source of West Eurasian ancestry of Roma. Onge represented the South Asian source of Roma ancestry. ROLLOFF analysis, which is based on the exponential decay rate of admixture LD (Fig. 5), estimated that the date of the beginning of gene flow between West Eurasians and Roma occurred 29.883 +/− 2.353 generations ago, which means that Roma admixture with West Eurasians began approximately 800–935 years ago, taken into account that one generation equals 29 years [25]. According to the calculated weighted LD values (Fig. 6), analysis with ALDER gave similar results, ALDER dates this event to 28.45 +/−2.66 generations, 750–900 years ago.

### The source of European and South Asian Ancestry of Roma

Previous tests were able to refer only to the West Eurasian ancestry of Roma, which includes also their admixture with West Eurasians before their exodus and during their migration period (populations of the Caucasus region, from the Middle East and also Central Asia) besides their admixture with Europeans. Using identity-by-descent segment analysis, we investigated the relationship of Europeans and the Roma. In order to find the South Asian origin of Romani people, we also investigated the IBD sharing between South Asians (Indian and Pakistani populations) and the Roma population.

**Fig. 7:**
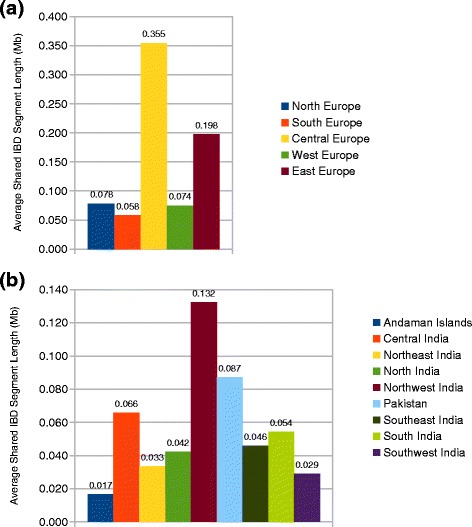
Population relationships based on identity-by-descent sharing estimation. We computed the genome-wide pairwise average shared IBD length between certain groups. **a** Average shared IBD length between Roma and Europeans. **b** Average shared IBD length between Roma and South Asians

IBD analysis showed that Roma are closer related to European populations (Fig. 7a). Central European populations show a significantly higher share in the European ancestry of Roma than other regions of Europe. Average shared IBD segment length of Roma with Central European populations was 0.355 Mb. Eastern nations of Europe also show higher IBD sharing with Roma. The average length of shared IBD segments was 0.058 Mb. These findings are consistent with the demographic data of Roma and the suggested migration route the Roma took during their migration from South Asia into Europe.

Analyzing the source of South Asian ancestry of Roma revealed that Roma shows the highest relatedness to Northwest Indian groups throughout India with an average shared IBD segment length value of 0.132 Mb. However, Pakistani groups show also high relatedness to Roma compared to other regions of India with an average share of 0.087 Mb (Fig. 7b). The extent of IBD share between Roma and Pakistani populations was only approached by the IBD share of Roma with Central Indian groups, which was 0.066 Mb.

### Estimated homozygozity by descent in Roma

**Fig. 8:**
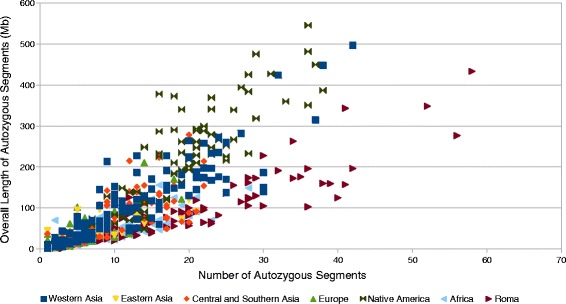
Individual genome-wide HBD estimation in Roma and HGDP populations. Grouping of HGDP populations are based on their regional location. Each point represents one individual. Color coding of Roma and HGDP groups are described in the figure legend

As previous genome-wide SNP data based studies suggested, Roma population has descended from a small number of ancestors due to one or more founder events before reaching Europe. To estimate the extent of HBD segments in Roma using our extended Roma dataset, we applied also Refined IBD. We compared Roma to other worldwide populations using the HGDP data. We separated the HGDP dataset to regional groups as Europe, Africa, Americas (Native American groups), Western Asia, Eastern Asia and Central and Southern Asia. We found that Roma have the highest individual genome-wide HBD compared to worldwide populations, and also observed that individuals in our extended Roma dataset shows an even more high HBD than results of previous studies suggested. Native American and Western Asian individuals from the HGDP data showed a slightly similar extent of HBD (Fig. 8).

## Discussion

Population structure and ancestry estimation analyses using PCA and model-based clustering methods placed Roma between Europeans and South Asians. These analyses anticipated that European (or more precisely West Eurasian) ancestry in Roma is significant and its proportion is higher than the proportion of the Indian ancestry. Our results showed that Northwest and likely Central Indians are the closest to Roma from Indian groups and Pakistani groups might also play an important role in the South Asian ancestry of Roma.

We confirmed with formal test of admixture that Roma are a mixture of West Eurasians and South Asians as ancestry analyses suggested and proportion of West Eurasian Ancestry in Roma compared to Onge (as accurate surrogate for a South Asian ancestral group) was also estimated. The proportion of West Eurasian ancestry in Roma was approximately 81.08%, which value corresponded to the previously reported results.

We estimated the date of the West Eurasian admixture of Roma, which corresponds to the date of previous reports based on genome-wide marker data and also to historical data, which state that Romani arrived to the Balkans in the 11th and 12th centuries [2]. Admixture of Roma with West Eurasian populations occurred 750–900 years ago.

Using our extended datasets of Roma and Indian samples and involving also Pakistani samples in our tests, we performed IBD analysis. Our results suggest that the source of South Asian ancestry of Roma could expand to the Pakistani area. Our results showed an even greater involvement of Northwest Indian populations in the South Asian ancestry of the Romani people.

We also measured the individual genome-wide HBD in Roma compared to worldwide populations. Using significantly higher number of Roma individuals, which gives us a more representative sample size of Romani people, individual genome-wide HBD showed an even more degree than the results of previous studies suggested.

Using genome-wide SNP array data of extended number of Roma individuals and Indian groups confirmed that the South Asian source of Roma ancestry originates likely from the Northwest region of India, with a less significant involvement of Central India. Investigated Northwest and Central Indian ethnic groups were the Meghawal, Gujarati, Bhil, Jain, Gond, Kharia and Satnami. However, the area of origin might also extend to the region of Pakistan, the neighboring country of India, since Pakistani populations Balochi, Brahui, Burusho, Kalash, Makrani, Pashtun and Sindhi showed a significant relatedness to Romani people according to our analyses. We estimated that the West Eurasian ancestry of Roma originates mainly from East and Central European populations, represented here with Bosnians, Croatians, Czechs, Hungarians, Polish, Romanians, Serbians, Slovakians, Russian and Ukrainian. These data corresponds to the demographic data of European Roma.

## Conclusion

Using a uniquely high number of Roma samples and Indian groups allowed us to further investigate the ancestry of Romani people. This study aimed to refine the findings of previous studies that investigated the history of Roma based on genome-wide SNP array data.

In conclusion, the results of our study suggest that the West Eurasian ancestry of Roma originates likely from Central and East Europe, and Northwest India plays an even more important role in the South Asian ancestry of Roma than previous studies suggested. Our results also suggest that besides Northwest Indian populations, Pakistani populations play also an important role of the source of South Asian ancestry of Romani people. These new findings extend the South Asian origin of the Romani people making the Pakistani region a similarly important source of ancestry for the Romani people as the Indian subcontinent.

## Additional files

Additional file 1: ADMIXTURE analysis of Roma, Europeans and South Asian populations. ADMIXTURE analysis results with K = 3 to K = 8 hypothetical ancestral groups. Cross-validation error was the lowest at K = 5. Each column represents one individual and each column group refers to a certain ethnic group labeled on the bottom of the figure. (PDF 151 kb)

Additional file 2: Results of D-statistics. Investigating whether Roma have both West Eurasian and South Asian ancestry using the D-statistics algorithm of ADMIXTOOLS 1.1 Software Package. Applied unrooted phylogenetic trees were ((CEU, CHB)(Roma, Onge)), ((CEU, YRI)(Roma, Onge)), ((TSI, CHB)(Roma, Onge)) and ((TSI, YRI)(Roma, Onge)). (XLSX 10 kb)

Additional file 3: Estimating the genome-wide proportion of West Eurasian and South Asian ancestry of Roma. (XLSX 12 kb)

Additional file 4: Residual fit from trees estimated by TreeMix. The residuals visualization of the ML trees shown on Fig. 4. (PDF 45 kb)
