# Supplementary material for: Refining the South Asian Origin of the Romani people
Source: BMC Genet. 2017 Aug 31;18:82. doi: 10.1186/s12863-017-0547-x (PMC5580230; doi:10.1186/s12863-017-0547-x)
Supplement: Supplementary file 1 — ADMIXTURE analysis of Roma, Europeans and South Asian populations. ADMIXTURE analysis results with K = 3 to K = 8 hypothetical ancestral groups. Cross-validation error was the lowest at K = 5. Each column represents one individual and each column group refers to a certain ethnic group labeled on the bottom of the figure. (PDF 151 kb) [file 12863_2017_547_MOESM1_ESM.pdf]

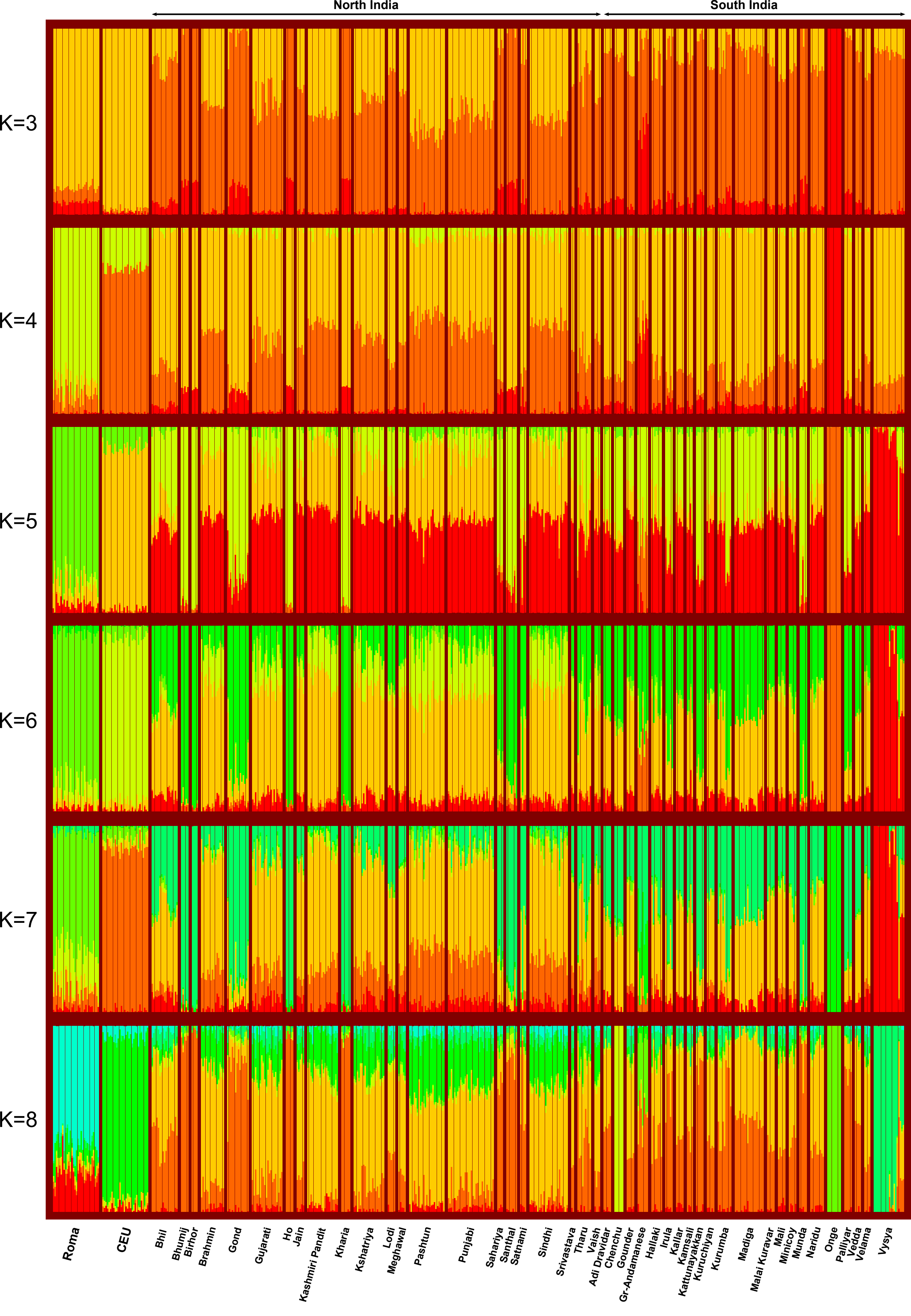

CEU - Utah residents with Northern and Western European ancestry from the CEPH collection.
