# Supplementary material for: Refining the South Asian Origin of the Romani people
Source: BMC Genet. 2017 Aug 31;18:82. doi: 10.1186/s12863-017-0547-x (PMC5580230; doi:10.1186/s12863-017-0547-x)
Supplement: Supplementary file 4 — Residual fit from trees estimated by TreeMix. The residuals visualization of the ML trees shown on Fig. 4. (PDF 45 kb) [file 12863_2017_547_MOESM4_ESM.pdf]

(a)

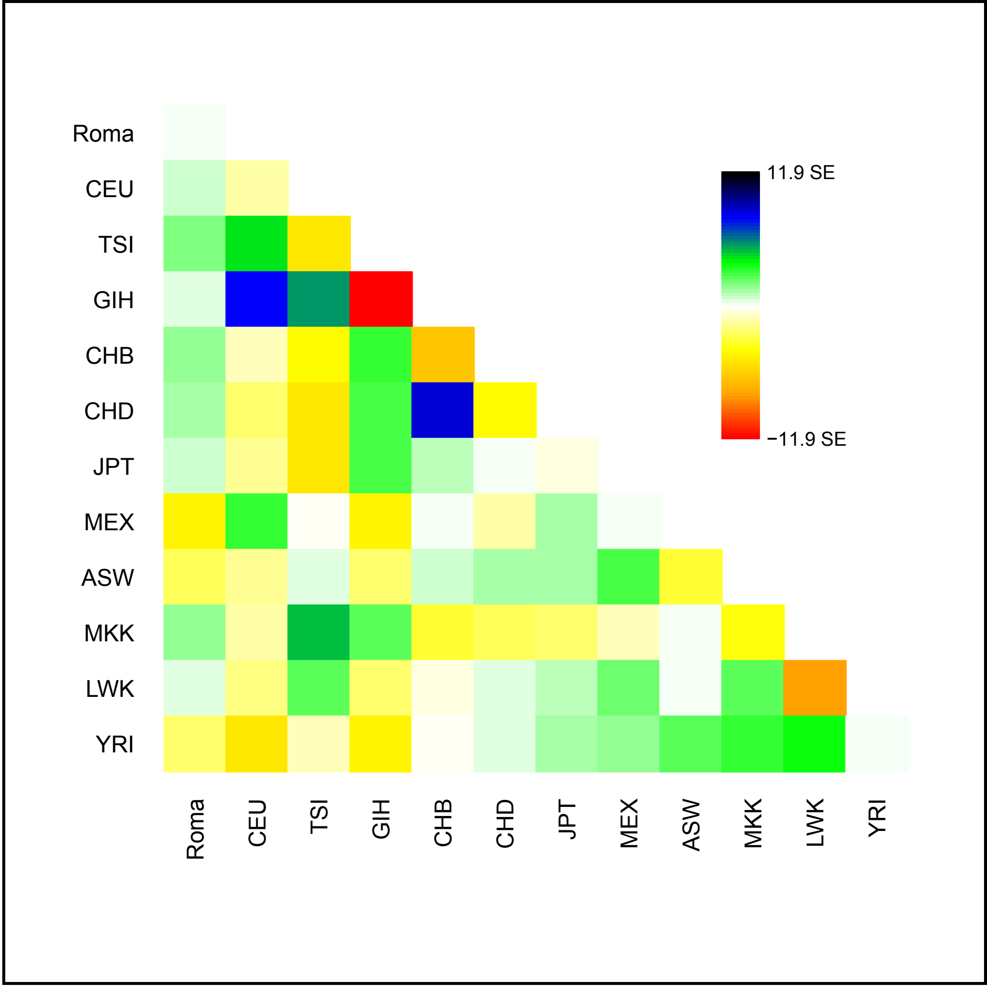
Roma
CEU
TSI
GIH
CHB
CHD
JPT
MEX
ASW
MKK
LWK
YRI
11.9 SE
−11.9 SE

(b)

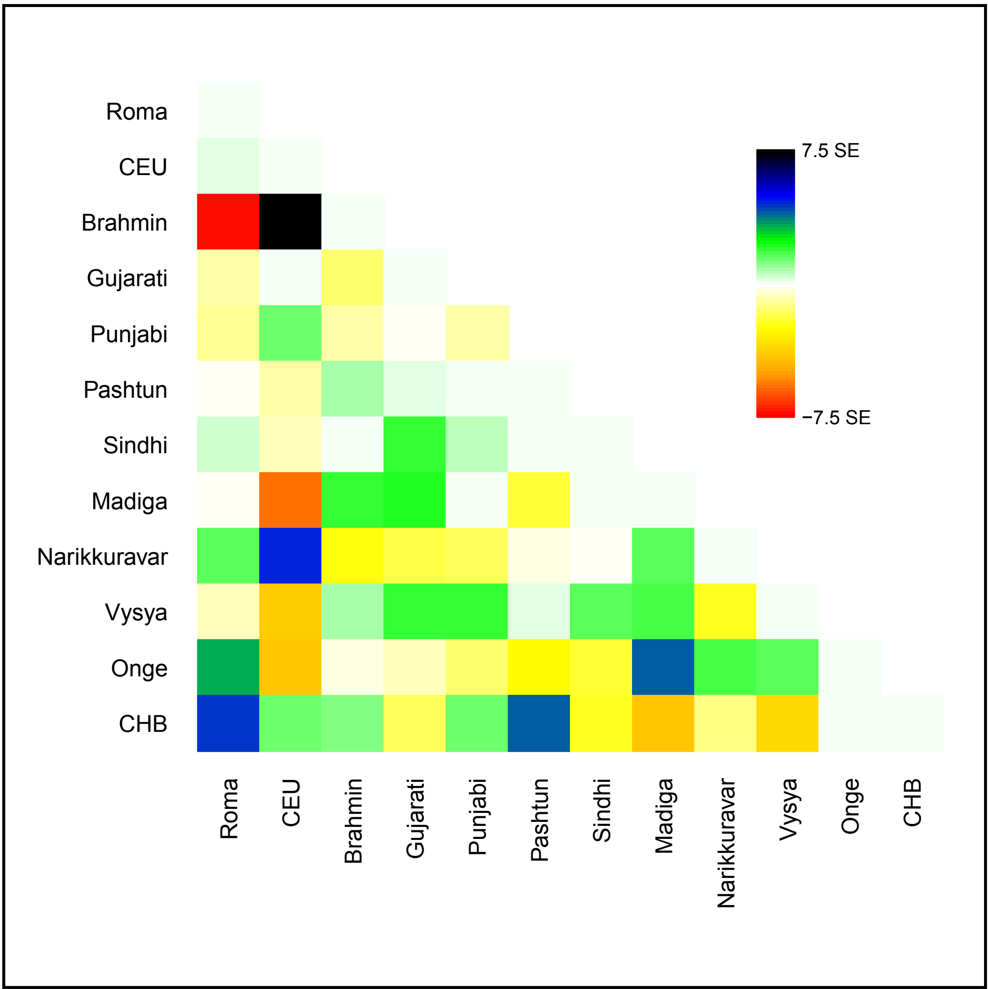
Roma
CEU
Brahmin
Gujarati
Punjabi
Pashtun
Sindhi
Madiga
Narikkuravar
Vysya
Onge
CHB
7.5 SE
−7.5 SE
